# The contagious impact of playing violent video games on aggression: Longitudinal evidence

**DOI:** 10.1002/ab.21857

**Published:** 2019-08-13

**Authors:** Tobias Greitemeyer

**Affiliations:** ^1^ Department of Psychology University of Innsbruck Innsbruck Austria

**Keywords:** aggression, contagion, longitudinal data, violent video games

## Abstract

Meta‐analyses have shown that violent video game play increases aggression in the player. The present research suggests that violent video game play also affects individuals with whom the player is connected. A longitudinal study (*N* = 980) asked participants to report on their amount of violent video game play and level of aggression as well as how they perceive their friends and examined the association between the participant's aggression and their friends’ amount of violent video game play. As hypothesized, friends’ amount of violent video game play at Time 1 was associated with the participant's aggression at Time 2 even when controlling for the impact of the participant's aggression at Time 1. Mediation analyses showed that friends’ aggression at Time 1 accounted for the impact of friends’ amount of violent video game play at Time 1 on the participant's aggression at Time 2. These findings suggest that increased aggression in video game players has an impact on the player's social network.

## 1 INTRODUCTION

Given its widespread use, the public and psychologists alike are concerned about the impact of violent video game play. In fact, a great number of studies have addressed the effects of exposure to violent video games (where the main goal is to harm other game characters) on aggression and aggression‐related variables. Meta‐analyses have shown that playing violent video games is associated with increased aggression in the player (Anderson et al., 2010; Greitemeyer & Mügge, 2014). The present longitudinal study examines the idea that violent video game play also affects the player's social network, suggesting that concern about the harmful effects of playing violent video games on a societal level is even more warranted.

### 1.1 Theoretical perspective

When explaining the effects of playing violent video games, researchers often refer to the General Aggression Model (GAM) proposed by Anderson & Bushman (2002). According to this theoretical model, person and situation variables (sometimes interactively) may affect a person's internal state, consisting of cognition, affect, and arousal. This internal state then affects how events are perceived and interpreted. Based on this decision process, the person behaves more or less aggressively in a social encounter. For example, playing violent video games is assumed to increase aggressive cognition and affect, which in turn results in behavioral aggression. An extension of this model further assumes that increased aggression due to previous violent video game play may instigate an aggression escalation cycle in that the victim also behaves aggressively (cf. Anderson & Bushman, 2018, Figure 5). The present research tested key predictions derived from the GAM and its extension, that (a) violent video game play is associated with increased aggression in the player and that (b) individuals who are connected to the player will also become more aggressive.

### 1.2 Effects of violent video game play on aggression

The relationship between violent video game play and aggression has been examined in studies employing cross‐sectional, longitudinal, and experimental designs. Cross‐sectional correlational studies typically show a positive relationship between the amount of violent video game play and aggression in real‐world contexts (e.g., Gentile, Lynch, Linder, & Walsh, 2004; Krahé & Möller, 2004). Several longitudinal studies have been conducted, showing that habitual violent video game play predicts later aggression even after controlling for initial aggressiveness (e.g., Anderson, Buckley, & Carnagey, 2008). That violent video game play has a causal impact on aggression and related information processing has been demonstrated by experimental work (e.g., Anderson & Carnagey, 2009; Gabbiadini & Riva, 2018). Finally, meta‐analyses corroborated that violent video game play significantly increases aggressive thoughts, hostile affect, and aggressive behavior (Anderson et al., 2010; Greitemeyer & Mügge, 2014). Some studies failed to find significant effects (e.g., McCarthy, Coley, Wagner, Zengel, & Basham, 2016). However, given that the typical effect of violent video games on aggression is not large, it is to be expected that not all studies reveal significant effects.

### 1.3 The contagious effects of aggression

Abundant evidence has been collected that aggression and violence can be contagious (Dishion, & Tipsord, 2011; Huesmann, 2012; Jung, Busching, & Krahé, 2019). Indeed, the best predictor of (retaliatory) aggression is arguably previous violent victimization (Anderson et al., 2008; Goldstein, Davis, & Herman, 1975). However, even the observation of violence can lead to increased violence in the future (Widom, 1989). Overall, it is a well‐known finding that aggression begets further aggression. Given that violent video game play increases aggression, it thus may well be that this increased aggression then has an impact on people with whom the player is connected.

Correlational research provides initial evidence for the idea that the level of people's aggression is indeed associated with how often their friends play violent video games (Greitemeyer, 2018). In particular, participants who did not play violent video games were more aggressive the more their friends played violent video games. However, due to the cross‐sectional design, no conclusions about the direction of the effect are possible. It may be that violent video game players influence their friends (social influence), but it is also conceivable that similar people attract each other (homophily) or that there is some shared environmental factor that influences the behavior of both the players and their friends (confounding). That is, it is unclear whether indeed aggression due to playing violent video games spreads or whether the effect is reversed, such that aggressive people are prone to befriend others who are attracted to violent video game play. Moreover, it is possible that some third variable affected both, participants’ reported aggression and their friends’ amount of violent video game play. There is also the possibility that people are unsure about the extent to which their friends play violent video games. In this case, they may perceive their friends as behaving aggressively and then (wrongly) infer that the friends play violent video games. To disentangle these possibilities and to show that the effect of violent video game play (i.e., increased aggression in the player) indeed has an impact on the player's social network, relationships among variables have to be assessed over time while covarying prior aggression (Bond & Bushman, 2017; Christakis & Fowler, 2013).

Verheijen, Burk, Stoltz, van den Berg, and Cillessen (2018) tested the idea that players of violent video games have a long‐term impact on their social network. These authors found that participants’ exposure to violent video games increased their friend's aggressive behavior 1 year later. However, given that the authors did not examine whether the violent video game player's increased aggression accounts for the impact on their friend's aggressive behavior, it is unknown whether violent video game play indeed instigates an aggression cycle. For example, players of violent video games may influence their friends so that these friends will also play violent video games. Any increases in aggression could then be an effect of the friends playing violent video games on their own.

### 1.4 The present research

The present study examines the longitudinal association between the participant's aggression and their friends’ amount of violent video game play, employing an egocentric networking approach (Stark & Krosnick, 2017). In egocentric networking analyses, participants provide self‐reports but also report on how they perceive their friends. In the following, and in line with Greitemeyer (2018), the friends were treated as the players and the participant was treated as their friends’ social network. Please note that ties between the participant's friends (i.e., whether friends also know each other) were not assessed (Greitemeyer, 2018; Mötteli & Dohle, 2019), because this information was not needed for testing the hypothesis that participants become more aggressive if their friends play violent video games. It was expected that friends’ amount of violent video game play at Time 1 would predict the participant's aggression at Time 2 even when controlling for the impact of the participant's aggression and amount of violent video game play at Time 1. It was further examined whether friends’ aggression at Time 1 would account for the impact of friends’ amount of violent video game play at Time 1 on the participant's aggression at Time 2. Such findings would provide suggestive evidence that violent video game play may instigate an aggression cycle. The study received ethical approval from the Internal Review Board for Ethical Questions by the Scientific Ethical Committee of the University of Innsbruck. The data and materials are openly accessible at https://osf.io/jp8ew/.

## 2 METHOD

### 2.1 Participants

Participants were citizens of the U.S. who took part on Amazon Mechanical Turk. Because it was unknown how many of the participants will complete both questionnaires, no power analyses were conducted a priori but a large number of participants was run. At Time 1, there were 2,502 participants (1,376 females, 1,126 males; mean age = 35.7 years, *SD = *11.8). Of these, 980 participants (522 females, 458 males; mean age = 38.9 years, *SD = *12.5) completed the questionnaire at Time 2. Time 1 and Time 2 were 6 months apart. There were no data exclusions, and all participants were run before any analyses were performed. The questionnaire included some further questions (e.g., participant's perceived deprivation) that are not relevant for the present purpose and are reported elsewhere (Greitemeyer & Sagioglou, 2018).^1^ Given that the questionnaire was relatively short, no attention checks were employed.

### 2.2 Procedure and measures

Procedure and measures were very similar to Greitemeyer (2018), with the main difference that individuals participated at two time points (instead of one). After providing demographics, self‐reported aggressive behavior was assessed. As in previous research (e.g., Krahé & Möller, 2010), participants indicated for 10 items how often they had shown the respective behavior in the past 6 months. Sample items are: “I have pushed another person” and “I have spread gossip about people I don't like” (5 items each address physical aggression and relational aggression, respectively). All items were rated on a scale from 1 (*never*) to 5 (*very often*), and scores were averaged. Participants were then asked about their amount of violent video game play, employing one item: “How often do you play violent video games (where the goal is to harm other game characters)?” (1 = *never* to 7 = *very often*).

Afterwards, participants learned that they will be asked questions about people they feel closest to. These may be friends, coworkers, neighbors, relatives. They should answer questions for three contacts with whom they talked about important matters in the last few months. For each friend, they reported the level of aggression (αs between = 0.90 and 0.91) and the amount of violent video game play, employing the same questions as for themselves. Responses to the three friends were then averaged. Finally, participants were thanked and asked what they thought this experiment was trying to study, but none noted the hypothesis that their friend's amount of violent video game play would affect their own level of aggression. At Time 2, the same questions were employed. Reliabilities for how participants perceived the level of aggression for each friend were between 0.89 and 0.90.

## 3 RESULTS

Descriptive statistics, intercorrelations, and internal consistencies of all measures are shown in Table 1.

**Table 1:** Means, standard deviations, and bivariate correlations

	*M*	*SD*	1	2	3	4	5	6	7	8
1. Participant's amount of violent video game play (T1)	2.74	2.09	—							
2. Participant's aggression (T1)	1.38	0.52	.15	.89						
3. Friends’ amount of violent video game play (T1)	2.28	1.31	.59	.18	.44					
4. Friends’ aggression (T1)	1.39	0.49	.14	.69	.25	.76				
5. Participant's amount of violent video game play (T2)	2.50	1.93	.83	.12	.55	.12	—			
6. Participant's aggression (T2)	1.30	0.45	.13	.50	.18	.43	.14	.88		
7. Friends’ amount of violent video game play (T2)	2.18	1.27	.55	.18	.69	.22	.61	.22	.51	
8. Friends’ aggression (T2)	1.33	0.44	.13	.40	.19	.51	.13	.74	.25	.79

*Note*: For Time 1, *N* = 2,502; for Time 2, *N* = 980. All correlation coefficients: *p* < .001. Where applicable, α reliabilities are presented along the diagonal.

### 3.1 Time 1 (*N* = 2,502)

The relationship between the amount of violent video game play and reported aggression was significant, both for the participant and the friends. That is, violent video game play was associated with increased aggression in the player and participants perceived their friends who play more violent video games to be more aggressive than their less‐playing friends. Participant's and friends’ amount of violent video game play as well as their level of reported aggression, respectively, were also positively associated, indicating that participants perceived their friends to be similar to them. Most importantly, participant's aggression was significantly associated with friends’ amount of violent video game play.^2^

**Figure 1:**
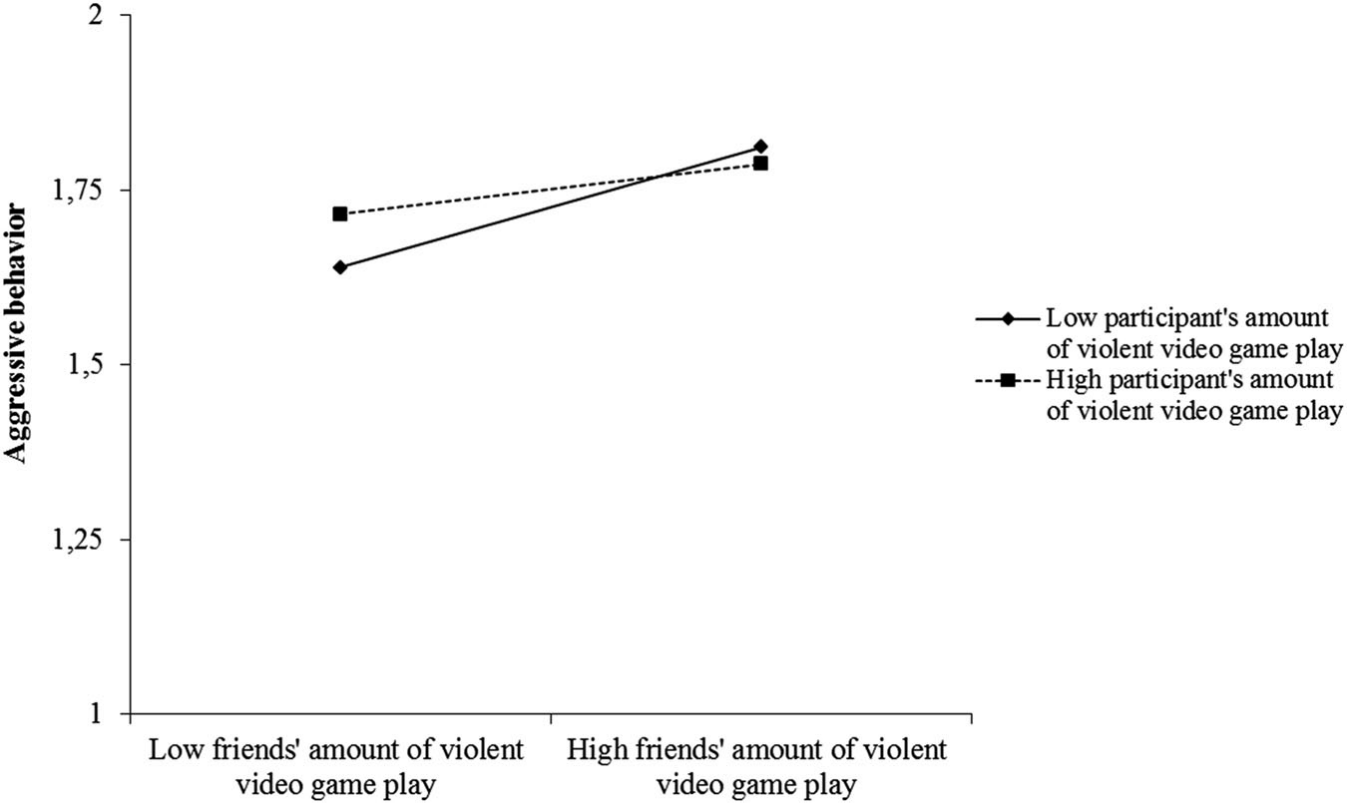
Simple slopes of the interactive effect of friends’ amount of violent video game play and the participant's amount of violent video game play on the participant's aggression, controlling for participant sex and age (Time 1, *N* = 2,502)

It was then examined whether friends’ amount of violent video game play is still associated with the participant's aggression when controlling for the participant's amount of violent video game play. Participant sex (coded 1 = male, 2 = female) and age were included as covariates. In fact, a bootstrapping analysis showed that the impact of friends’ amount of violent video game play remained significant (point estimate = 0.08, *SE* = 0.02, *t* = 4.72, *p* < .001, 95% confidence interval [CI] = [0.05, 0.11]). Participant's amount of violent video game play (point estimate = 0.03, *SE* = 0.01, *t* = 2.18, *p* = .029, 95% CI = [0.00, 0.05]) and the interaction were also significant (point estimate = −0.01, *SE* = 0.00, *t* = 2.41, *p* = .016, 95% CI = [−0.02, −0.00]). At low levels of the participant's amount of violent video game play (− 1 SD, equals that the participant does not play violent video games in the present data set), friends’ amount of violent video game play was associated with the participant's aggression (point estimate = 0.07, *SE* = 0.01, *t* = 5.06, *p* < .001, 95% CI = [0.04, 0.10]). At high levels of the participant's amount of violent video game play ( + 1 SD), friends’ amount of violent video game play was also associated with the participant's aggression (point estimate = 0.03, *SE* = 0.01, *t* = 3.14, *p* = .002, 95% CI = [0.01, 0.06]), but the effect was less pronounced. Participants were thus most strongly affected by whether their social network plays violent video games when they do not play violent video games themselves (Figure 1). Participant sex was not significantly associated with the participant's aggression (point estimate = −0.04, *SE* = 0.02, *t* = 1.95, *p* = .052, 95% CI = [−0.09, 0.00]), whereas age was (point estimate = −0.01, *SE* = 0.00, *t* = 7.84, *p* < .001, 95% CI = [−0.009, −0.005]).

### 3.2 Time 1 and Time 2 (*N* = 980)

**Figure 2:**
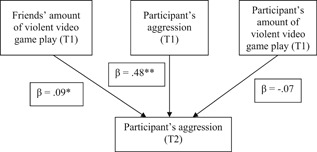
Participant's aggression at Time 2 simultaneously predicted by friends’ amount of violent video game play, participant's aggression, and participant's amount of violent video game play at Time 1. Participant sex and age were controlled for, but were not included in the figure (see the main text for the impact of participant sex and age). *p < .01, ** *p* < .001 (*N* = 980)

To examine the impact of friends’ amount of violent video game play on the participant's aggression over time, a cross‐lagged regression analysis was performed on the data. Participant's amount of violent video game play, friends’ amount of violent video game play, participant's aggression at Time 1, as well as participant sex and age were used as predictors for participant's aggression at Time 2. The overall regression was significant, *F*(5,974) = 68.92, *R* ^2^ = 0.26, *p* < .001. Most importantly, friends’ amount of violent video game play at Time 1 significantly predicted participant's aggression at Time 2, *t* = 2.60, *β* = .09, 95% CI = (0.02, 0.16), *p* = .009. Participant's aggression showed high stability, *t* = 16.77, *β* = .48, 95% CI = (0.42, 0.53), *p* < .001, whereas the participant's amount of violent video game play at Time 1 did not significantly predict the participant's aggression at Time 2, *t* = 1.77, *β* = −.07, 95% CI = (− 0.14, 0.01), *p* = .077 (Figure 2).^3^, ^4^ Participant sex also received a significant regression weight, *t* = 2.08, *β* = −.06, 95% CI = (−0.12, −0.00), *p* = .038, whereas age did not, *t* = 1.93, *β* = −.06, 95% CI = (−0.12, 0.00), *p* = .054. The reverse effect that the participant's aggression at Time 1 predicts their friends’ amount of violent video game play at Time 2 when controlling for the participant's amount of violent video game play and friends’ amount of violent video game play at Time 1, as well as participant sex and age, was not significant, *t* = 0.67, *β* = .02, 95% CI = (−0.03, 0.06), *p* = .504.

**Figure 3:**
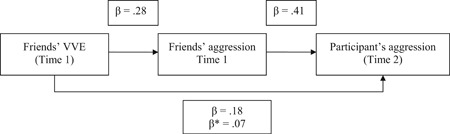
Mediation of the impact of friends’ violent video game exposure (VVE) at Time 1 on the participant's aggression at Time 2 by friends’ aggression at Time 1. All paths are significant. β* = the coefficient from friends’ VVE at Time 1 to the participant's aggression at Time 2 when controlling for friends’ aggression at Time 1 (*N* = 980)

Finally, it was examined whether the impact of friends’ amount of violent video game play at Time 1 on the participant's aggression at Time 2 would be mediated by friends’ level of aggression at Time 1 (while controlling for the participant's aggression and amount of violent video game play at Time 1 as well as participant sex and age). A bootstrapping analysis (with 5.000 iterations) showed that the impact of friends’ level of aggression at Time 1 on the participant's aggression at Time 2 was significant (point estimate = 0.16, *SE* = 0.04, *t* = 4.28, *p* < .001, 95% CI = [0.09, 0.23]). Participant's aggression at Time 1 was also a significant predictor (point estimate = 0.34, *SE* = 0.03, *t* = 10.19, *p* < .001, 95% CI = [0.27, 0.40]). Friends’ amount of violent video game play at Time 1 (point estimate = 0.03, *SE* = 0.01, *t* = 1.82, *p* = .069, 95% CI = [−0.00, 0.05]) and participant's amount of violent video game play at Time 1 (point estimate = −0.01, *SE* = 0.01, *t* = 1.65, *p* = .099, 95% CI = [−0.03, 0.00]) were not significant predictors. Participant sex significantly predicted the participant's aggression at Time 2 (point estimate = −0.06, *SE* = 0.03, *t* = 2.31, *p* = .021, 95% CI = [−0.11, −0.01]), whereas age did not (point estimate = −0.00, *SE* = 0.00, *t* = 1.90, *p* = .058, 95% CI = [−0.00, 0.00]). The indirect effect was significantly different from zero (point estimate = 0.01, 95% CI = [.00, 0.02]), suggesting that participants are more aggressive if their friends play violent video games for the reason that these friends are more aggressive. Figure 3 displays a simplified version of this mediation effect, based on regression coefficients and without controlling for the participant's aggression at Time 1, the participant's amount of violent video game play at Time 1, participant sex, and age.

## 4 DISCUSSION

Violent video games have an impact on the player's aggression (Anderson et al., 2010; Greitemeyer & Mügge, 2014), but—as the present study shows—they also increase aggression in the player's social network. In particular, participants who do not play violent video games reported to be more aggressive the more their friends play violent video games. Mediation analyses showed that the increased aggression in the friends accounted for the relationship between friends’ amount of violent video game play and the participant's aggression. Because changes in aggression over time were assessed, the present study provides evidence for the hypothesized effect that violent video game play is associated with increased aggression in the player, which then instigates aggression in their social network. Importantly, the impact of the participant's amount of violent video game play was controlled for, indicating that the relationship between friends’ amount of violent video game play and the participant's aggression is *not* due to the friends being similar to the participants. Moreover, the reverse effect that aggressive people will become attracted to others who play violent video games was not reliable. The present research thus documents the directional effects that violent video games is associated with increased aggression in the player and that this increased aggression then has an impact on people with whom the player is connected.

Overall, the present study provides comprehensive support for key hypotheses derived from the GAM and its extension (Anderson & Bushman, 2018). It shows that violent video game play is associated with increased aggression in the player and it documents that others who are connected to players might be also affected even when controlling for their own amount of violent video game play. To the best of my knowledge, this study is the first that shows that because violent video game players are more aggressive their friends will become aggressive, too. Previous research either employed a cross‐sectional design and thus could not address the direction of the effect (Greitemeyer, 2018) or did not examine whether the effect of violent video game play (i.e., increased aggression) indeed spreads (Verheijen et al., 2018). As proposed by the GAM and its extension (Anderson & Bushman, 2018), increased aggression in violent video game players appears to instigate an aggression escalation cycle (cf. Anderson et al., 2008).

It is noteworthy, however, that the longitudinal effect of the participant's amount of violent video game play at Time 1 on the participant's aggression at Time 2 was not reliable. Hence, although there were significant correlations between participants’ aggression and their violent video game use at both time points, the present study does not show that repeatedly playing violent video games leads to long‐term changes in aggression. However, a recent meta‐analysis of the long‐term effects of playing violent video games confirmed that violent video game play does increase physical aggression over time (Prescott, Sargent, & Hull, 2018), although the effect size was relatively small (*β* = 0.11) and thus single studies that produce nonsignificant results are to be expected. Importantly, in the present study, a single‐item measure of violent video game play was employed. In contrast, previous research on the relationship between violent video game play and the player's aggression has often employed multi‐item measurement scales that are typically more reliable and precise (for an overview, Busching et al., 2015). Hence, it may well be that due to the limitations of the single‐item measure of the participant's amount of violent video game play the relationship between participants’ violent game play and their aggressive behavior was artificially reduced.

Even though the longitudinal design allows ruling out a host of alternative explanations for the impact of violent video games on the player's social network, causality can only inferred by using an experimental design. Future research may thus randomly assign participants to play a violent or nonviolent video game (players) and assesses their aggression against new participants (partners). It can be expected that the partners suffer more aggression when the player had played a violent, compared to a nonviolent, video game. Afterwards, it could be tested whether the partner of a violent video game player is more aggressive than a partner of a nonviolent video game player. Given that the partner is not exposed to any video games, firm causal conclusions could be drawn that violent video game play affects aggression in people who are connected to violent video game players. It could be also tested whether the partner of a violent video game player would not only be more likely to retaliate against the player, but also against a third party. In fact, previous research into displaced aggression has shown that people may react aggressively against a target that is innocent of any wrongdoing after they have been provoked by another person (Marcus‐Newhall, Pedersen, Carlson, & Miller, 2000). It may thus well be that the effect of playing violent video games spreads in social networks and that even people who are only indirectly linked to violent video game players are affected.

An important limitation of the present egocentric network data is the reliance on the participant's perception of their social network, leaving the possibility that participants did not accurately perceive their friends. It is noteworthy that participants perceived their friends to be highly similar to them. In this regard, it is important to keep in mind that participants always provided self‐ratings first, followed by perceptions of their friends. It is thus conceivable that participants used their self‐ratings as anchors for the perceptions of their friends. Such a tendency, however, would reduce the unique effect of friends’ amount of violent video game play on the participant's aggression when controlling for the participant's amount of violent video game play. The finding that participants in particular who do not play violent video games reported to be more aggressive if their friends play violent video games also suggests that the impact of violent video games on the player's social network is not due to participants providing both self‐reports and how they perceive their friends. Finally, rather than by their friends’ objective qualities, people's behavior should be more likely to be affected by their subjective perceptions of their friends.

As noted in the introduction, participants may not be aware of the extent to which their friends play violent video games and hence used the perception of how aggressive their friends are as an anchor for estimating their friends’ amount of violent video game play. Importantly, however, the participant's aggression at Time 2 was significantly predicted by friends’ amount of violent video game play at Time 1 even when controlling for friends’ level of aggression at Time 1 (see Figure 3). Moreover, whereas aggression might be used for estimating violent video game exposure of the friends, participants should be well aware of the extent to which they play violent video games so that anchoring effects for participant's self‐reports are unlikely. However, given that it cannot be completely ruled out that the correlation between violent game play of friends at Time 1 and aggressive behavior of participants at Time 2 reflects a pseudocorrelation that is determined by the correlation between aggressive behavior of friends at Time 1 and aggressive behavior of the participant at Time 2, future research that employs sociocentric network analyses where information about the friends is provided by the friends themselves would be informative.

Another limitation is the employment of self‐report measures to assess aggressive behavior. Self‐report measures are quite transparent, so participants may have rated themselves more favorably than is actually warranted. In fact, mean scores of reported aggressive behavior were quite low. This reduced variance, however, typically diminishes associations with other constructs. In any case, observing how actual aggressive behavior is influenced by the social network's violent video game play would be an important endeavor for future work. It also has to be acknowledged that some participants may have reported on different friends at Time 1 and Time 2. Future research would be welcome that ensures that participants consider the same friends at different time points.

Future research may also shed some further light on the psychological processes. In the present study, the violent video game players’ higher levels of aggression accounted for the relationship between their amount of violent video game play and the participants’ reported aggression. It would be interesting to examine why the players’ aggression influences the aggression level of their social network. One possibility is that witnessing increased aggression by others (who play violent video games) leads to greater acceptance of norms condoning aggression, which are known to be an antecedent of aggressive behavior (Huesmann & Guerra, 1997). After all, if others behave aggressively, why should one refrain from engaging in the same behavior.

Another limitation of the present work is that it was not assessed *how* participants and their friends play violent video games. A recent survey (Lenhart, Smith, Anderson, Duggan, & Perrin, 2015) showed that many video game users play video games together with their friends, either cooperatively or competitively. This is insofar noteworthy as there might be some overlap between participants’ and their friends’ violent video game play. Moreover, cooperative video games have been shown to increase prosocial tendencies (Greitemeyer, 2013; Greitemeyer & Cox, 2013; but see Verheijen, Stoltz, van den Berg, & Cillessen, 2019) and decrease aggression (Velez, Greitemeyer, Whitaker, Ewoldsen, & Bushman, 2016). In contrast, competitive video game play increases aggressive affect and behavior (e.g., Adachi & Willoughby, 2016). Hence, future research should examine more closely whether participants play violent video games on their own, competitively, or cooperatively. The latter may show some positive effects of video game play, both on the player and the player's friends, whereas opposing effects should be found for competitive video games.

To obtain high statistical power and thus to increase the probability to detect significant effects, data were collected via an online survey. The current sample was drawn from the MTurk population (for a review of the trend to rely on MTurk samples in social and personality psychology, see Anderson et al., 2019). Samples drawn from MTurk are not demographically representative of the U.S. population as a whole. For example, MTurk samples are disproportionally young and female and they are better educated but tend to be unemployed (for a review, Keith, Tay, & Harms, 2017). On the other hand, MTurk samples are more representative of the U.S. population than are college student samples (Paolacci & Chandler, 2014) and the pool of participants is geographically diverse. Moreover, MTurk participants appear to be more attentive to survey instructions than are undergraduate students (Hauser & Schwarz, 2016). Nevertheless, future research on the impact of violent video game play on the player's social network that employs other samples would improve the generalizability of the present findings.

In conclusion, violent video game play is not only associated with increased aggression in the player but also in the player's social network. In fact, increased aggression due to violent video game play appears to instigate further aggression in the player's social network. This study thus provides suggestive evidence that not only players of violent video games are more aggressive, but also individuals become more aggressive who do not play violent video games themselves but are connected to others who do play.

## ENDNOTES

1 Participant's perceived deprivation was positively related to both violent video game exposure, *r*(2,502) = 0.08, *p* < .001, and reported aggression, *r*(2,502) = 0.14, *p* < .001. However, the relationship between violent video game exposure and reported aggression, *r*(2,502) = 0.15, *p* < .001, was relatively unaffected when controlling for perceived deprivation, *r*(2,499) = 0.14, *p* < .001.

2 Given that the measures of violent video game exposure and aggressive behavior violated the normal distribution, Spearman's *ρ* coefficients were also calculated. However, the pattern of finding was very similar (e.g., the crucial relationship between the participant's aggression and friends’ amount of violent video game play was 0.18 [Pearson] and 0.17 [Spearman]). All these analyses can be obtained from the author upon request.

3 When dropping friends’ amount of violent video game play from the analysis, the participant's amount of violent video game play at Time 1 still did not predict participant's aggression at Time 2, *t* = 0.44, *β* = −.01, 95% CI = (− 0.02, 0.01), *p* = .657 (when controlling for participant's aggression at Time 1, participant sex, and age).

4 Given that violent video games primarily model physical aggression, violent video games should have a stronger effect on the player's physical aggression than on other types of aggression. In fact, the impact of the participant's amount of violent video game play at Time 1 on the participant's physical aggression at Time 2, *t* = 1.49, *β* = .04, 95% CI = (− 0.00, 0.02), *p* = .136 (when controlling for the participant's physical aggression at Time 1), was more pronounced than the impact on the participant's relational aggression at Time 2, *t* = 0.52, *β* = .02, 95% CI = (− 0.01, 0.02), *p* = .603 (when controlling for the participant's relational aggression at Time 1), but both effects were not significant.
